# *Staphylococcus aureus*-Specific Tissue-Resident Memory CD4^+^ T Cells Are Abundant in Healthy Human Skin

**DOI:** 10.3389/fimmu.2021.642711

**Published:** 2021-03-16

**Authors:** Astrid Hendriks, Malgorzata Ewa Mnich, Bruna Clemente, Ana Rita Cruz, Simona Tavarini, Fabio Bagnoli, Elisabetta Soldaini

**Affiliations:** ^1^GSK, Siena, Italy; ^2^Medical Microbiology, University Medical Center Utrecht, Utrecht University, Utrecht, Netherlands

**Keywords:** *Staphylococcus aureus*, infection, immunity, human skin, tissue-resident memory T cells, CD4^+^ T cells

## Abstract

The skin is an immunocompetent tissue that harbors several kinds of immune cells and a plethora of commensal microbes constituting the skin microbiome. *Staphylococcus aureus* is a prominent skin pathogen that colonizes a large proportion of the human population. We currently have an incomplete understanding of the correlates of protection against *S. aureus* infection, however genetic and experimental evidence has shown that CD4^+^ T cells play a key role in orchestrating a protective anti-*S. aureus* immune response. A high *S. aureus*-specific memory CD4^+^ T cell response has been reported in the blood of healthy subjects. Since T cells are more abundant in the skin than in blood, we hypothesized that *S. aureus*-specific CD4^+^ T cells could be present in the skin of healthy individuals. Indeed, we observed proliferation of tissue-resident memory CD4^+^ T cells and production of IL-17A, IL-22, IFN-γ and TNF-β by cells isolated from abdominal skin explants in response to heat-killed *S. aureus*. Remarkably, these cytokines were produced also during an *ex vivo* epicutaneous *S. aureus* infection of human skin explants. These findings highlight the importance of tissue-resident memory CD4^+^ T cells present at barrier sites such as the skin, a primary entry site for *S. aureus*. Further phenotypical and functional characterization of these cells will ultimately aid in the development of novel vaccine strategies against this elusive pathogen.

## Introduction

The skin provides a physical and immunological barrier for invading pathogens, while also maintaining symbiotic interactions with skin commensals. There are numerous specialized immune cells present in the skin that maintain skin homeostasis and act as the first line of defense against pathogens. It has been estimated that human skin contains roughly twice as many memory T cells than blood (1). Different memory T cell subsets can be phenotypically identified in human skin based on the presence of surface markers and the capacity to emigrate and enter the circulation. Tissue resident memory T (Trm) cells are a subset of memory T cells phenotypically and functionally distinct from their circulating counterparts (1, 2). In particular, human skin-resident memory T (Tsrm) cells can be identified through the surface expression of the skin-homing marker cutaneous lymphocyte-associated antigen (CLA), the memory T cell marker CD45RO and the tissue-retention marker CD69. CLA binds selectively and avidly to the vascular lectin E-selectin while CD69 prevents sphingosine-1-phosphate receptor 1 mediated egress from tissues into the circulation (3). Skin-resident T cell memory has been observed in response to *Candida albicans, Leishmania major*, Herpes simplex virus as well as commensal bacteria. Most importantly, Tsrm cells contribute to localized protection against re-infection with cutaneous pathogens (4–9). In addition, Trm cell development has been tracked in mice following vaccination and was positively correlated with vaccination efficacy (10–13), making Trm cells a promising target for vaccination (14–19).

The Gram-positive bacterium *Staphylococcus aureus* is the leading cause of skin and soft tissue infections globally (20). In addition, the rapid emergence of antibiotic resistance has highlighted the need for alternative treatments such as vaccination to combat *S. aureus* infections. However, to design an efficacious vaccine, it is important to have a complete understanding of the correlates of protection against this pathogen, which is currently lacking (21, 22).

Based on data from mouse and human studies, there is a general consensus that CD4^+^ T cells, and in particular Th17 and Th1 subsets, contribute to protective immunity against *S. aureus* infection (23–26). Furthermore, healthy individuals have a considerable number of circulating memory CD4^+^ T cells specific for *S. aureus*, likely due to repeated encounters over time with this skin pathobiont (27, 28). However, to our knowledge, the existence of *S. aureus*-specific tissue resident memory CD4^+^ T cells in healthy human skin has not yet been addressed.

Using human skin explants, which represent a valuable model to study skin-resident immune responses of human skin to microbes (29), we here show that *S. aureus*-specific CD4^+^ Tsrm cells are commonly found in the skin of healthy individuals. This finding uncovers CD4^+^ Tsrm cells as previously neglected cellular players in the cutaneous human immune response to *S. aureus* and thus may aid in the development of novel vaccine strategies against *S. aureus* SSTIs.

## Materials and Methods

### Preparation of Single Cell Suspensions From Human Skin Explants

**Figure 1:**
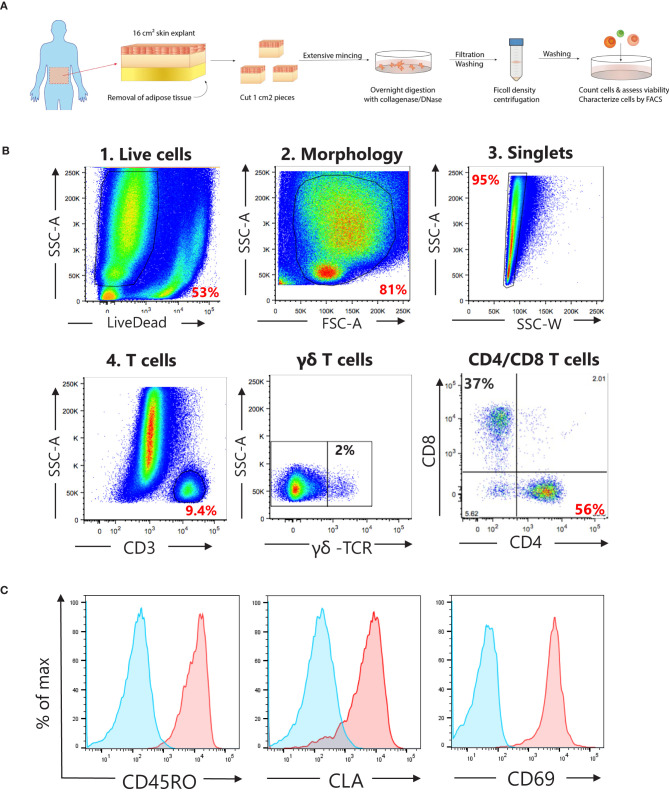
Characterization of T cell subsets in cell suspensions obtained from human skin explants. **(A)** Schematic overview of the protocol used to obtain single cell suspensions from human skin explants. **(B)** Gating strategy to analyze T cell subsets, γδ-, CD4^+^, and CD8^+^ T cells, in single cell suspensions. **(C)** Representative histograms (in red) showing the surface expression for skin resident memory T cells markers, CD45RO (memory), CLA (skin homing), and CD69 (tissue retention) on live CD4^+^ cells. Blue histograms represent unstained cells.

Fresh human skin explants (16 cm^2^) derived from abdominoplasty surgical waste of healthy women (age 40 ± 11, body mass index 25 ± 3) were purchased from Biopredic (France). Explants were shipped at 4°C and received within 48 h following the surgery. Upon arrival, samples were immediately processed as shown in Figure 1A. In short, adipose tissue was removed with dissection scissors followed by additional scraping with a disposable scalpel (Swann-Morton, Sheffield). Skin was cut in 1 cm^2^ pieces, washed repeatedly with PBS and incubated for 1 h at RT in RPMI 1640 (Invitrogen) containing 1 mg/ml collagenase type 1 (Life technologies). Next, skin pieces were extensively minced with disposable scalpels and incubated overnight at 37°C at 5% CO_2_ in a 6-well plate with 1 mg/ml collagenase type 1 (Life technologies) and 20 μg/ml DNAse (Sigma) in 5 ml c-RPMI [RPMI 1640 containing penicillin-streptomycin-glutamine, sodium pyruvate, minimum essential medium non-essential amino acids (all from Gibco), and 10% heat-inactivated FBS (Hyclone)]. The next day, the skin cell suspension was pipetted vigorously, pooled and filtered sequentially through a 100 μm and a 40 μm cell strainers (Corning). Skin debris was further removed by Ficoll-Paque Premium (GE Healthcare) gradient separation. The viability (83% ± 6.5) and the cell yield (0.63 ± 0.33 × 10^6^ cells/cm^2^ skin) of the obtained single cell suspensions were assessed with a Vi-CELL XR cell counter (Beckman Coulter).

### Heat-Killed (HK) Microbes

Methicillin resistant *S. aureus* USA300 LAC strain and the coagulase negative staphylococci *S. epidermidis* 1457 strain and *S. lugdunensis* SL13 strain were grown to mid-exponential phase (OD_600_ 0.6) (30, 31). Next, bacteria were washed with PBS to remove secreted proteins, resuspended in sterile PBS, plated on Tryptic Soy Agar (TSA) for CFU counts and inactivated in a dry block heater at 90°C for 45 min. After inactivation bacteria were washed three times with PBS and protein content was measured using the Pierce™ BCA Protein Assay kit (Thermo Scientific). Samples concentrations were adjusted to 25 μg/ml, which corresponds to ~1 × 10^8^ CFU/ml (25). Bacterial killing was verified by plating the HK bacteria for 5 days on TSA. Heat-killed (HK) bacteria were aliquoted and stored at −20°C. HK *S. epidermidis* (FDA strain PCI 1200), HK *C. albicans* (ATCC 10231), and HK *E. coli* (O111:B4) were purchased from Invivogen.

### CD4^+^ T Cell Proliferation and Cytokine Production in Response to HK Microbes by Click-iT EdU/V-PLEX Assay

Single cell suspensions obtained from the skin explants were seeded at 500,000 live cells/well for all conditions but CD3/CD28 (for which half the number of cells were plated) in a final volume of 200 μl c-RPMI in round-bottom 96 wells plates (Corning). Cells were rested for at least 24 h at 37°C with 5% CO_2_ to restore surface marker expression and reduce cellular stress due to the isolation procedure. Cell culture medium was replaced with: (1) fresh medium alone (no stimulation, negative control) or containing: (2) 10^6^ CFU HK microbes corresponding to a multiplicity of infection of 2; (3) Tetanus toxoid (5 μg/ml, Novartis); (4) anti-CD28 (2 μg/ml, clone CD28.2, BD Bioscience, cat # 555725) added to anti-CD3 (1 μg/ml, clone OKT3, BD Bioscience, cat # 566685) coated wells (CD3/CD28, polyclonal stimulation, positive control). After 3 days of culture the thymidine analog EdU (10 μM) was added to the cultures for the last 16 h. At day four, cell culture supernatants were collected and stored at −20°C for cytokine analysis while CD4^+^ T cell proliferation was assessed by Click-iT EdU assay (Click-iT Plus EdU Alexa Fluor 488 Flow cytometry assay kit, Invitrogen), as recently described (Clemente et al., manuscript in preparation). Cytokines were measured using the 27-V-PLEX human kit (MesoScale Discovery) following manufacturer's instructions. Plates were analyzed by a MESO Quickplex SQ 120 reader and cytokine concentrations were determined using MSD discovery workbench 4.0. Values below or above the detection limits were given the value of ½ LLOD (Lower Limit Of Detection) or 2x ULOD (Upper Limit Of Detection), respectively. Cytokines that were consistently above or below the detection limits, or showed no differences across all stimuli were excluded from further analysis.

### Flow Cytometry

For the phenotypic characterization of T cell subsets in the single skin cell suspensions, cells were stained with Live/Dead Near-IR Dead cell stain kit (Invitrogen) for 20 min at room temperature (RT), washed and blocked with 2% rabbit serum in PBS on ice for 20 min. Next, cells were stained for CD4, CD8, γδ-TCR, CD45RO, CLA, and CD69 for 20 min at 4°C, washed with PBS, and fixed with Cytofix (BD Bioscience). Gating strategy is shown in Figure 1B.

For T cell proliferation experiments, after surface staining with CD4, CD8, CD45RO and CLA and fixation, cells were permeabilized with PBS 1% BSA, 0.5% saponin for 30 min at 4°C, washed with PBS 1% BSA, 0.5% saponin followed by the Click-iT reaction (Click-iT Plus EdU Alexa Fluor 488 Flow cytometry assay kit, Invitrogen). After 30 min at RT, cells were washed with PBS 1% BSA, 0.5% saponin and stained for CD3 for 15 min at RT. After two washes, the cells were analyzed on a BD LSR II flow cytometer, and data was analyzed using FlowJo 10 (TreeStars). All antibodies used in this study are shown in Supplementary Table 1.

### Cytokine Production in Response to *ex vivo* Epicutaneous *S. aureus* Infection of Human Skin Explants

Infection of human skin explants was performed according to a previously described protocol (32). In short, after removal of adipose tissue, the skin sheet was pinned in a dissection board and stripped 30 times with a hypoallergenic tape (Transpore, 3M). Eight mm punch biopsies were collected using disposable biopsy punches (Kai Medical). The punches were washed with culture medium (Advanced DMEM; Gibco) once, followed by two washes with PBS to remove antibiotics. Next, the punches were placed in 12-well transwell plates with 0.4 μm pore size (Corning), containing 1 ml of culture medium. Finally, the punches were infected in duplicate with USA300 LAC strain (5 × 10^6^ CFU in 1 μl PBS) and cultured at air-liquid interface for 2, 24, or 72 h at 37°C, 5% CO_2_. At each indicated time point, culture supernatants were collected, filtered and stored at −20°C for cytokine analysis that was performed using the 27-V-PLEX human kit (MesoScale Discovery).

### Statistical Analysis

GraphPad Prism 8.0.1 was used to perform statistical analysis. Data were analyzed using one-way ANOVA with Dunnett's test, paired Wilcoxon test or paired *t*-test, as indicated. Significant differences (*p* < 0.05) are shown.

## Results

### Tissue-Resident Memory CD4^+^ T Cells Present in the Skin of Healthy Subjects Proliferate in Response to *S. aureus*

**Figure 2:**
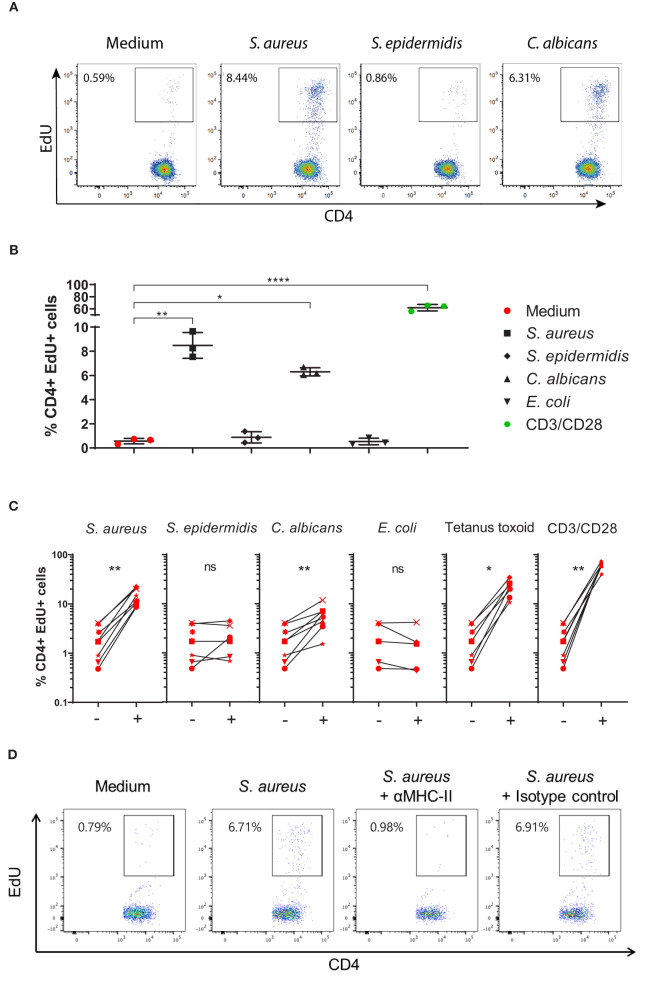
Tissue-resident memory CD4^+^ T cells present in the skin of healthy subjects proliferate in response to *S. aureus*. **(A)** Representative dot-plots showing CD4^+^EdU^+^ cells in cell cultures, obtained from a skin explant of an healthy subject, stimulated for 4 days with heat-killed (HK): *S. aureus* USA300 LAC strain, *S. epidermidis* PCI 1200 strain, *C. albicans*, or left unstimulated (medium). **(B)** Reproducibility of the Click-iT EdU assay. Representative results showing the percentages of CD4^+^EdU^+^ T cells obtained from triplicate skin cell cultures from the healthy subject shown in **(A)** in response to different stimuli. ^*^p < 0.05, ^**^p < 0.01, ^****^p < 0.0001 as assessed by one-way ANOVA. **(C)** Proliferation of CD4^+^ Tsrm cells from skin explants of 8 healthy subjects in response to different HK microbes, Tetanus toxoid, anti-CD3/anti-CD28 antibodies or medium alone. Average percentages of CD4^+^EdU^+^ cells of each of the 8 subjects analyzed, in triplicate, are shown by an identifying symbol. Per donor, each stimulated group, indicated by a +, was compared to the non-stimulated group (medium), indicated by a –, by paired Wilcoxon test, ^*^p < 0.05, ^**^p < 0.01. **(D)** Representative dot plots showing proliferating CD4^+^ Tsrm cells (CD4^+^EdU^+^) after 4-day culture in medium alone (negative control) or with HK *S. aureus* alone or in combination with MHC class-II blocking antibodies or isotype control antibodies.

To investigate if healthy human skin contains CD4^+^ Tsrm cells specific for *S. aureus*, we stimulated single cell suspensions prepared from skin explants from eight healthy donors with heat-killed (HK) *S. aureus*. The vast majority (>90%) of the isolated CD4^+^ T cells had a Tsrm phenotype based on the surface expression of the memory marker CD45RO, the skin-tropic marker CLA and the tissue-retention marker CD69 (Figure 1C). After 4 day stimulation with HK microbes, we identified CD4^+^ T cells that have neo-synthesized DNA by the flow cytometry-based Click-iT EdU proliferation assay (CD4^+^EdU^+^ cells, Figures 2A,B). These cells were CD4^+^ Tsrm based on their expression of CD45RO and CLA (Supplementary Figure 1). As shown in Figure 2C, the analysis of 8 healthy subjects showed a statistically significant CD4^+^ Tsrm cell proliferation in response to HK *S. aureus* but not to HK *S. epidermidi*s, which is a major component of the human skin microbiome (33). In agreement with previous studies, we observed specific CD4^+^ Tsrm cell proliferation in response to *C. albicans* (Figure 2C) (6), while no proliferation was observed in response to *E. coli*, which is not part of the skin microbiome (Figure 2C). Interestingly, a strong proliferation of CD4^+^ Tsrm cells was also induced by the recall antigen Tetanus toxoid (Figure 2C) that commonly induces a strong T cell response in human blood (27, 34). Polyclonal T cell stimulation with anti-CD3/CD28 antibodies, which was used as positive control, induced the strongest CD4^+^ Tsrm cell proliferation in all donors as expected (Figure 2C).

Heat-inactivated intact bacteria have been described to be devoid of superantigens, which stimulate T cells in a non-specific manner (25, 35). To further prove that the observed CD4^+^ Tsrm cell proliferation was antigen-specific, we added MHC class-II blocking antibodies or the isotype control to the skin cell cultures. Indeed, in the presence of MHC-II blocking antibodies, CD4^+^ T cell proliferation in response to HK *S. aureus* was abolished while the isotype control had no effect (Figure 2D). In addition, no CD4^+^ T cell proliferation was detected by Click-iT EdU assay upon stimulation of peripheral blood mononuclear cells (PBMCs) of some healthy subjects with HK *S. aureus*, while a strong proliferation was observed in response to the staphylococcal T cell superantigen SEB, as expected (Supplementary Figure 2A).

To further assess the staphylococcal species-specificity on CD4^+^ Tsrm cell proliferation, we analyzed the proliferative response to the coagulase-negative *S. lugdunensis*, which is also a skin commensal (33). Analysis of cells obtained from explants from five healthy subjects showed no proliferation in response to either *S. lugdunensis* SL13 strain or *S. epidermidis* 1457 strain while proliferation to *S. aureus* USA300 LAC strain was confirmed (Supplementary Figure 2B). Taken together, these findings support the presence of *S. aureus*-specific CD4^+^ tissue-resident memory T cells in healthy human skin.

### Cells Isolated From the Skin of Healthy Subjects Produce Pro-inflammatory Cytokines in Response to *S. aureus*

**Figure 3:**
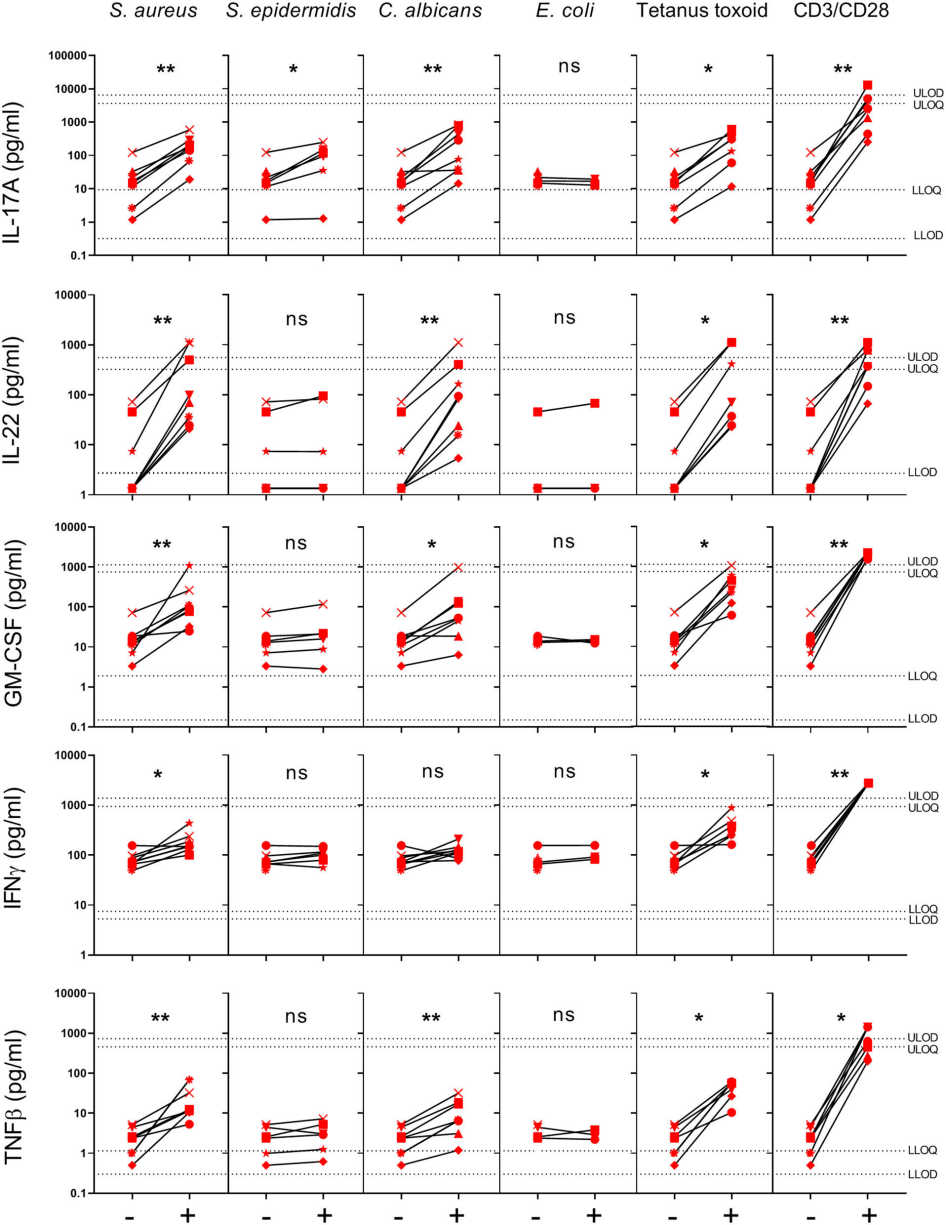
Cytokines secreted in the supernatants of skin cell cultures stimulated with different HK microbes. Supernatants were collected from samples used to determine CD4^+^ Tsrm cell proliferation by the Click-iT EdU assay. Cytokines were quantified by V-PLEX assays. Cytokine production in response to the different stimuli is shown for 8 donors (indicated by individual symbols), except for Tetanus toxoid (7), *S. epidermidis* (6) and *E. coli* (3). Per donor, each stimulated group, indicated by a +, was compared to the non-stimulated group (medium), indicated by a –, by paired Wilcoxon test, ^*^p < 0.05, ^**^p < 0.01. LLOD, Lower limit of detection; ULOD, upper limit of detection; LLOQ, lower limit of quantification; ULOQ, upper limit of quantification.

To further investigate the response of healthy human skin to *S. aureus*, we analyzed the cytokine profile in the supernatants of *S. aureus*-specific CD4^+^ Tsrm cells analyzed by Click-iT EdU assay (Figure 2C), collected after 4 days of stimulation, by 27-V-PLEX. As shown in Figure 3, significant increases in production of IL-17A (141.20 vs. 12.34 pg/ml), IL-22 (131.67 vs. 4.22 pg/ml), IFN-γ (176.61 vs. 78.82 pg/ml), GM-CSF (138.73 vs. 13.34 pg/ml), and TNF-β (18.48 vs. 1.84 pg/ml) were observed in response to HK *S. aureus* stimulation while HK *S. epidermidis* induced a significant increase in IL-17A production only (48.29 vs. 12.34 pg/ml). Interestingly, *C. albicans*, as well as Tetanus toxoid and the polyclonal stimulation with anti-CD3/CD28 antibodies induced the same pattern of cytokines as *S. aureus* while in response to *E. coli* none of the analyzed cytokines was induced. Remarkably, the production of IL-17A, IL-22, IFN-γ, GM-CSF, and TNF-β in response to HK *S. aureus* stimulation were substantially decreased by MHC class II blocking antibodies, indicating that CD4^+^ Tsrm cells were a major source of these cytokines (data not shown). In addition we obtained direct evidence of IL-17A and IL-22 production by CD4^+^EdU^+^ Tsrm cells by intracellular cytokine staining (data not shown).

### *Ex vivo* Epicutaneous *S. aureus* Infection of Healthy Human Skin Induces Pro-inflammatory Cytokines

**Figure 4:**
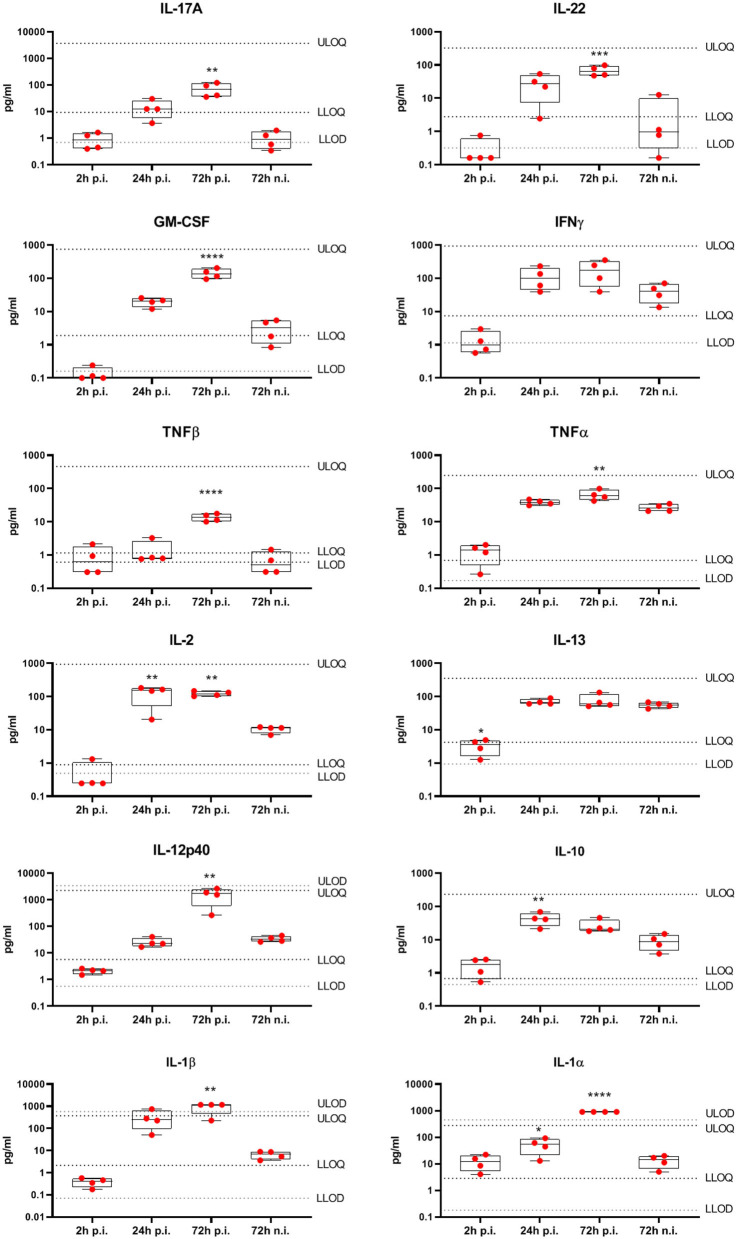
Cytokines produced by human skin from healthy subjects in response to *ex vivo* epicutaneous *S. aureus* USA300 LAC infection. Eight mm biopsy punches, prepared from tape stripped human skin explants from four donors, were put in transwells at air-liquid interface and infected epicutaneously with 5 × 10^6^ CFU *S. aureus* USA300 LAC strain in duplicate, or were left non-infected (n.i.) for 2, 24, or 72 h when culture medium was collected. Cytokine concentrations were assessed by V-PLEX assay. Box-and-whiskers extend from the 25th to 75th percentiles and the line inside the box represents the median. LLOD, Lower limit of detection; ULOD, upper limit of detection; LLOQ, lower limit of quantification; ULOQ, upper limit of quantification. Differences between cytokine concentrations measured at 2, 24, 72 hpi were compared with non-infected control at 72 h using an one-way ANOVA, ^*^p < 0.05, ^**^p < 0.01, ^***^p < 0.001, ^****^p < 0.0001.

To understand the local immune response to *S. aureus* within healthy human skin, we used an *ex vivo* epicutaneous infection model (32). Skin explants were tape-stripped to remove the *stratum corneum*, followed by topical application of 5 × 10^6^ CFU *S. aureus* USA300 LAC strain. Cytokines were quantified in the skin explants culture media by 27-V-PLEX at different time-points post infection (p.i.). At 24 h p.i. only IL-10, IL-1α, and IL-2 were produced at significantly higher levels compared to the non-infected control while at 72 h p.i. also IL-17A, IL-22, IFN-γ, IL-1α, IL-1β, GM-CSF, IL-12p40, TNF-α, and TNF-β levels were increased (Figure 4). These data show that, while at an initial stage of *S. aureus* infection the cytokine response of the skin is limited, it becomes strongly proinflammatory at later stages of infection. Furthermore, the cytokines induced by stimulation of cells extracted from the skin with HK *S. aureus*, namely IL-17A, IL-22, GM-CSF, IFN-γ, and TNF-β, were induced also by epicutaneous infection of human skin with live *S. aureus*, strengthening the value of this *in vitro* model.

## Discussion

Here, we show that *S. aureus*-specific CD4^+^ tissue-resident memory T cells are abundant in the skin of healthy subjects. In particular, by the use of a novel assay, Click-iT EdU/V-PLEX, which allows simultaneous detection of CD4^+^ T cell proliferation and cytokine quantification in cell culture supernatants, we revealed that stimulation of cells isolated from abdominal skin explants with HK *S. aureus* USA300 LAC strain induced: (1) The proliferation of CD4^+^ T cells that were identified as skin-resident memory T (Tsrm) cells based on the expression of the CLA skin-homing, CD45RO memory, and CD69 tissue-retention markers. (2) The secretion of proinflammatory cytokines, namely IL-17A, IL-22, GM-CSF, IFN-γ, and TNF-β. Remarkably, neither proliferation of CD4^+^ Tsrm cells nor secretion of proinflammatory cytokines except for IL-17A was observed in response to the common skin commensal *S. epidermidis*.

Mechanisms enabling the host to mount protective immune responses against pathogens while establishing a privileged relationship with commensal bacteria have been intensively studied but still remain largely unknown. One important feature of commensal-specific immunity is its uncoupling from inflammation and the maintenance of tissue homeostasis (8). Cytokines play a key role not only in the promotion of skin inflammation but also in skin homeostasis. Cytokine analysis of culture medium of both isolated skin cells stimulated with HK *S. aureus* and human skin explants infected *ex vivo* with *S. aureus* showed the production of cytokines involved in skin inflammation and tissue repair. Genetic evidence has highlighted a key role for IL-17-mediated immunity in protection against *S. aureus* skin infection, but not invasive staphylococcal disease, similarly to what has been observed for *C. albicans* infections (36, 37). IL-17 enhances the recruitment of neutrophils, which can kill *S. aureus*, to the site of infection, and stimulates the production of antimicrobial peptides (AMPs) that can be directly bactericidal (38–40). IL-22 promotion of skin inflammation is well-established (41), however a role of IL-22 in skin homeostasis has also emerged. In particular, IL-22 has been shown to induce the proliferation of keratinocytes and AMPs production (42, 43). In addition, IL-22 induces MHC class II expression on keratinocytes thereby promoting the selective accumulation of commensal-induced IFN-γ producing CD4^+^ T cells within murine skin (44). TNF-β induces angiogenesis, thereby contributing to wound repair (45, 46). Interestingly, cytokine analysis of human skin explants infected epicutaneously with *S. aureus* revealed also a significant production of IL-1α and IL-1β (Figure 4). These results are in agreement with previous studies showing that these cytokines were produced by murine keratinocytes after an epicutaneous *S. aureus* challenge (47, 48). The lack of IL-1 production by isolated human skin cells stimulated with HK *S. aureus* could be due to the lack of secreted bacterial proteins, including alpha-toxin that has a prominent role in the induction of IL-1 production by keratinocytes (49).

The Click-iT EdU/V-PLEX assay does not allow the identification of the cellular source of the detected cytokines. However, since we observed that the polyclonal T cell stimulation with anti-CD3/CD28 antibodies induced the same cytokine profile as HK *S. aureus* stimulation, and MHC class II blocking antibodies substantially decreased the production of these cytokines in response to HK *S. aureus* stimulation (data not shown) it seems likely that CD4^+^ Tsrm cells are a major source of the observed cytokines (50), although cytokine production by non-classical T cells cannot be ruled out (51–53). In addition we obtained direct evidence of IL-17A and IL-22 production by CD4^+^EdU^+^ Tsrm cells by intracellular cytokine staining (data not shown). It should be noted that studies, performed both in human and murine skin, suggest that Trm cells accumulate in the skin as a function of the number of infectious and inflammatory events over time. Indeed, laboratory mice, like newborn, but not adult, humans lack effector-differentiated and peripherally distributed memory T cells, including Tsrm cells (54, 55). In mice, γδ T cells have been identified as key IL-17 producers upon *S. aureus* infection (52). However, it has been shown that following infection of laboratory mice with *C. albicans*, while the initial IL-17-producing cells were γδ T cells, at later times the majority of *C. albicans*-reactive IL-17-producing T cells were CD4^+^ Tsrm cells. Importantly, IL-17-producing CD4^+^ Trm cells that responded to *C. albicans* were identified in normal human skin (4). Similarly, since humans, unlike laboratory mice, naturally encounter *S. aureus* repeatedly over time, we hypothesize that CD4^+^ Tsrm cells are the primary source of IL-17 produced in response to this bacterium in human skin. Indeed, comparable levels of IL-17A were produced in response to HK *S. aureus* or *C. albicans* in our experiments. On the other hand, *S. epidermidis* colonization of mouse skin has been shown to induce IL-17A-producing CD8^+^ T cells restricted to non-classical MHC class I molecules and characterized by immunoregulatory and tissue-repair signatures, which home to the epidermis (9). These cells could be the source of IL-17A produced in response to HK *S. epidermidis* in our experiments, although further research is needed to address this point.

A major difference between *S. aureus* and *S. epidermidis* is the secretion of numerous virulence factors including proteases and toxins such as alpha-toxin, that can damage the skin epithelial integrity (32, 56). Our results suggest that in order to halt *S. aureus* invasion, the cutaneous immunity deploys CD4^+^ Tsrm cells that secrete several cytokines with proven anti-*S. aureus* and tissue-repair activities. Induction of such a mild anti-bacterial immune response might be a strategy to limit local infection and prevent systemic spread, promoting a long-lasting equilibrium between this pathobiont and the host. Interestingly, this could be achieved through alpha-toxin that has been shown to modulate mouse CD4^+^ T cell differentiation limiting Th1 while promoting Th17 responses (57). However, once the skin is breached, the local immunity is dampened or the bacterial load is exceedingly high, this local response is no longer sufficient to control *S. aureus* (37). Indeed, the importance of antibodies and Th1 cells in controlling systemic *S. aureus* infections has been highlighted (25, 58).

Interestingly, a recent paper showed that neonatal mouse skin colonization with *S. epidermidis* facilitated immune tolerance to this bacterium via the induction of regulatory T (Treg) cells (49). This was not the case for *S. aureus* that, through alpha-toxin mediated IL-1β production by myeloid cells, limited the development of *S. aureus*-specific Treg cells thus enhancing skin inflammation upon later-life exposure to *S. aureus* (49). Similarly, we cannot rule out the presence of *S. epidermidis*-specific Treg cells, which are known to proliferate under homeostatic conditions, in our experiments (6).

The immune response of the skin to *S. aureus* has been intensively investigated in a number of elegant mouse studies (19). However, the anatomical and immunological differences between murine and human skin together with the different composition and exposure to skin microbiome limit the translational value of the results obtained in mice (29, 59). As a more biologically relevant model, we used human skin explants generated as surgical waste from cosmetic surgery performed on the abdomen. Of note, although *Staphylococcus aureus* colonization is most consistently identified in humans in the anterior nares, colonization has also been reported at other body sites including axilla, inguinal and rectal areas (60). In addition, *S. aureus* was cultured from 30% of abdominal skin swabs from healthy subjects (61). Since variability was reported among different skin sites, as a consequence differences in the relative abundance in CD4^+^ Trm cells specific for *S. aureus* at different locations can be envisaged. Nevertheless, studies have shown that following skin infection, Tsrm cells can migrate out of the skin and populate distant skin sites thus forming global skin immunity (16, 62). Interestingly, *S. aureus*-specific CD4^+^ Trm cells have been identified in gut tissue of healthy individuals. These cells showed increased IL-17A and reduced IFN-γ production as compared to cells with similar reactivity present in the circulation (63), similarly to what we report here for *S. aureus*-specific CD4^+^ Tsrm cells. Indeed, this seems to be a common characteristic of barrier-protective Trm cells. In addition, since some inborn errors of IL-17 immunity predispose not only to skin but also to lung *S. aureus* infection (37), the presence of *S. aureus*-specific CD4^+^ Tsrm cells in the lungs and their phenotype should be assessed.

While numerous efforts have been made toward designing a vaccine against *S. aureus*, unfortunately to this date none have been successful (64). Perhaps the most fundamental reason explaining the past failures of *S. aureus* vaccines is the lack of a complete understanding of protective immunity. Our results enforce the conclusion that since the contribution of local immune memory within tissues is becoming evident, it should be evaluated in vaccination efficacy studies (50). Since a sizable percentage of people experiencing *S. aureus* SSTI has recurrent infections (22, 65), it will be very informative to analyze the CD4^+^ Tsrm response to *S. aureus* in this population. In addition, it would be interesting to investigate the *S. aureus*-specific CD4^+^ Tsrm response in patients with atopic dermatitis, a chronic and relapsing inflammatory skin disorder associated with skin barrier impairment and the predominant *S. aureus* colonization (66).

In summary, we describe a skin-resident memory CD4^+^ T cell population within healthy human skin that is specific for the human skin pathogen *S. aureus*. While further research is needed to better characterize the phenotype, the antigen-specificity and the protective potential of these cells, this finding highlights that skin-resident memory CD4^+^ T cells could be a powerful and exploitable arm of adaptive immunity against this elusive pathobiont.

## Data Availability Statement

The raw data supporting the conclusions of this article will be made available by the authors, without undue reservation.

## Ethics Statement

The studies involving human participants were reviewed and approved by French Ministry of Higher Education, Research and Innovation. The patients/participants provided their written informed consent to participate in this study.

## Author Contributions

AH, MEM, and ES were involved in designing the study and wrote the paper. AH, MEM, BC, and ARC performed experiments. ST and BC set up the Click-iT EdU assay. ST provided valuable technical support with flow cytometry. AH, MEM, BC, and ES analyzed the data. FB and ES supervised the project. All authors critically revised the manuscript and approved it before submission.

## Conflict of Interest

AH, MEM, and ARC are Ph.D. fellows and are enrolled in the Infection and Immunity Ph.D. program, part of the graduate school of Life Sciences at Utrecht University and participated in a post graduate studentship program at GSK. BC was a PhD student from the University of Siena funded by GSK. ST, FB, and ES are employees of the GSK group of companies. FB hold shares in the GSK group of companies and holds pending and issued patents (WO/2019/158537, WO/2015/144691, WO/2015/144653, WO/2015/144655, WO/2014/033190, WO/2014/033191, WO/2014/033192, WO/2014/033193, WO/2013/030378, and WO/2010/119343) on S. aureus vaccine formulations.
