# Supplementary material for: Staphylococcus aureus-Specific Tissue-Resident Memory CD4+ T Cells Are Abundant in Healthy Human Skin
Source: Front Immunol. 2021 Mar 16;12:642711. doi: 10.3389/fimmu.2021.642711 (PMC8008074; doi:10.3389/fimmu.2021.642711)
Supplement: Supplementary file 1 [file Data_Sheet_1.docx]

**Supplementary material**

**Supplementary Table 1. List of antibodies used in this study**

| Antibody | Supplier | Clone | Catalogue number |
| --- | --- | --- | --- |
| anti-human CD3-PE-CF594 | BD biosciences | UCHT1 | 562280 |
| anti-human CD4-BV421 | BD biosciences | RPA-T4 | 562424 |
| anti-human CD8-APC | Biolegend | RPA-T8 | 301014 |
| anti-human-γδ-TCR-PECy7 | Biolegend | B1 | 331222 |
| anti-human CLA-BV605 | BD biosciences | HECA-452 | 563960 |
| anti-human CD45RO-BV650 | BD biosciences | UCHL1 | 563750 |
| anti-human CD69-BUV737 | BD biosciences | FN50 | 564439 |
| anti-human MHC class II HLA-DR, DP, DQ blocking antibody | BD biosciences | Tu39 | 555556 |
| mouse IgG2a, κ Isotype Control for anti-MHC class II blocking antibody | BD biosciences | G155-178 | 554645 |


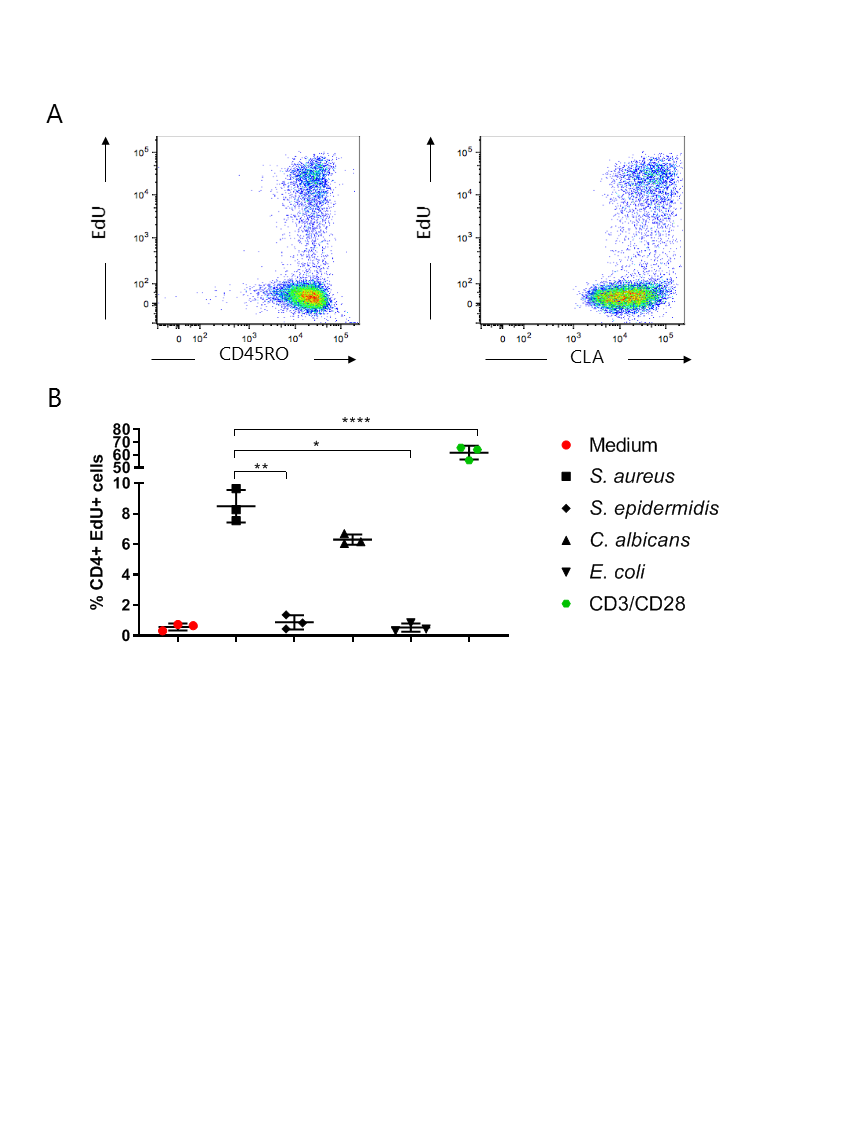


**Supplementary figure 1. Click-iT EdU assay tracks *S. aureus*-specific CD4^+^ Tsrm cell proliferation*.*** Representative dot plots showing surface expression of CD45RO and CLA on proliferating CD4^+^ T cells (CD4^+^EdU^+^), gated on live CD3^+^CD4^+^CD8^-^ cells.


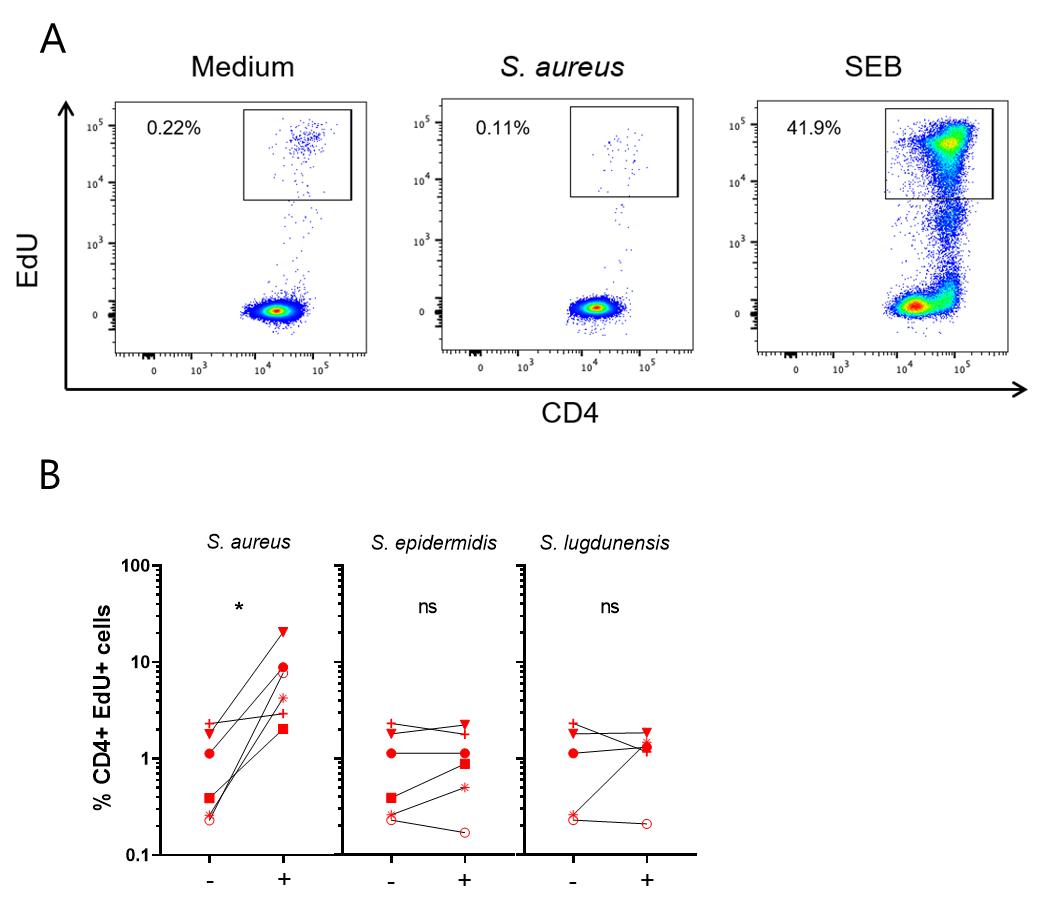


**Supplementary figure 2. Specificity of CD4^+^ Tsrm cell proliferation in response to *S. aureus*.** (A) Representative dot-plots showing CD4^+^EdU^+^ cells in PBMCs stimulated for 4 days with heat-killed (HK) *S. aureus*, the T cell superantigen *S. aureus* enterotoxin B (SEB, 1 µg/ml Sigma-Aldrich), or left unstimulated (medium). (B) CD4^+^ Tsrm proliferation in response to different HK staphylococcal species: *S. aureus* USA300 LAC, *S. epidermidis* 1457 strain, and *S. ludgunensis* SL13 strain. Per donor, each stimulated group, indicated by a +, was compared to the non-stimulated group (medium), indicated by a -, by paired Wilcoxon test, *p<0.05.
